# The impact of COVID-19 disease on the natural course of cirrhosis: Before and after starting vaccination

**DOI:** 10.3389/fmed.2022.1039202

**Published:** 2023-02-02

**Authors:** Onur Keskin, Hakan Oral, Tevhide Sahin, Taylan Kav, Erkan Parlak

**Affiliations:** ^1^Department of Gastroenterology, Hacettepe University School of Medicine, Ankara, Turkey; ^2^Department of Internal Medicine, Hacettepe University School of Medicine, Ankara, Turkey

**Keywords:** cirrhosis, COVID-19, death, vaccination, clinical course

## Abstract

**Background:**

Cirrhosis has been reported as an important risk factor for death in coronavirus disease 2019 (COVID-19) disease. In this study, we aimed to investigate the effects of COVID-19 on the natural course of cirrhosis before and after starting vaccination.

**Methods:**

The cirrhosis patients in our cohort (*n*: 140; median age:56; 71 female) were included in this study. The median MELD (Model For End-stage Liver Disease) score was 11 (6–25) and CCI (Charlson Comorbidity Index) score was 4 (1–11). In total, 85 had CTP (Child-Turcotte-Pugh)-A, 44 had CTP-B and 11 had CTP-C cirrhosis. The course of COVID-19 in this patient group was evaluated before and after COVID-19 vaccination.

**Results:**

Between March 2020 and January 2021, 36 of the 140 cirrhosis patients had developed COVID-19. Cirrhosis (+)/COVID-19 (+) and Cirrhosis (+)/COVID-19 (–) groups did not differ in terms of age, CCI and MELD-Na scores, or gender. There were six deaths in the Cirrhosis (+)/COVID-19 (+) group and five in the Cirrhosis (+)/COVID-19 (–) group [6/36 (16.6%) vs. 5/104 (4.8%); *p*: 0.03]. Patients who died were older, had higher CCI and MELD-Na scores, and lower albumin levels. Having had COVID-19 [6.45 (1.43–29.4); *p*: 0.015], higher MELD-Na score [1.35 (1.18–1.60); *p*: 0.001] and higher CCI score [1.65 (1.14–2.39); *p*: 0.008] were found to be independent predictors of mortality. After effective vaccination started in Turkey, only 11 of the remaining 129 patients developed COVID-19, and only one patient died, who was unvaccinated.

**Discussion:**

In our cirrhotic cohort, COVID-19 disease was associated with 16% mortality in the pre-vaccination period. COVID-19 vaccination prevents serious illness and death due to COVID-19 in cirrhotic patients.

## Introduction

The severe acute respiratory syndrome coronavirus-2 (SARS-CoV-2) and its infection coronavirus disease of 2019 (COVID-19) caused the death of many people all over the world in the past 2 years. Turkey has been one of the countries most affected by this virus. The virus can be fatal, especially in elderly individuals, by causing respiratory failure (1). The liver is also affected in COVID-19 infection. This affect ranges from simple transaminase elevations to the development of acute-on-chronic liver failure (ACLF) (2, 3).

It has been shown that bacterial and various viral infections have a worse course in cirrhosis patients compared to the normal population (4). The course of COVID-19 infection in patients with chronic hepatitis and cirrhosis has been shown in various studies. This situation is explained by the immune dysfunction that develops in cirrhosis. As a matter of fact, there are some studies showing that the presence of cirrhosis may adversely affect the course of COVID-19 infection (5). The development and widespread application of effective vaccines against COVID-19 infection all over the world have changed the course of the disease and reduced serious morbidity and mortality (6, 7). In this study, we aimed to compare the clinical course of cirrhosis patients with and without COVID-19 infection in the pre- and post-vaccination period.

## Materials and methods

### Patients

**FIGURE 1:**
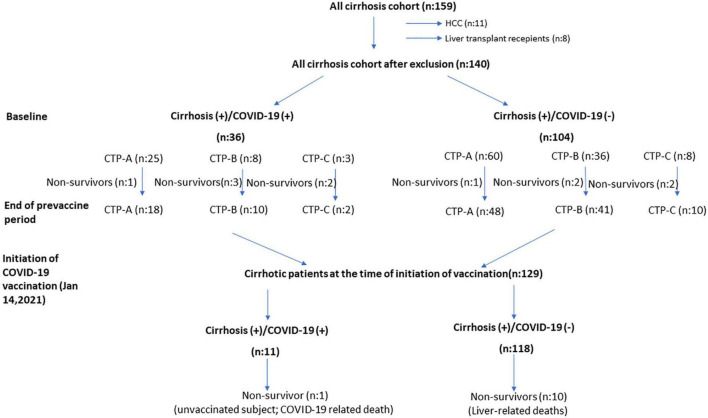
The course of cirrhosis patients during the 10 months pre-vaccine and 10 months post-vaccination periods.

After exclusion of patients with hepatocellular cancer (*n*: 11) and liver transplant recipients during follow-up (*n*: 8), all patients who were followed up with a diagnosis of cirrhosis before the pandemic (*n*: 140) in the Gastroenterology Department of Hacettepe University School of Medicine were included in the study (Figure 1). The patients were started to be followed on March 11, 2020, when the first COVID-19 case was seen in Turkey. During this 10 months pandemic period, the course of our cirrhotic patients was evaluated, and the follow-up data were obtained. In Turkey, vaccination against COVID-19 infection started with the inactivated whole-virion SARS-CoV-2 vaccine (Corona Vac) produced in China and was applied to healthcare workers and high-risk patients with chronic diseases. In our cirrhotic patient group, the course of COVID-19 infection was also evaluated in the period after the start of vaccination, that is, between January 14, 2021 and November 2021. Approval for the study was obtained from the Ethics Committee of Hacettepe University (GO 21/561).

The diagnosis of cirrhosis in these patients was made by clinical laboratory, imaging methods, endoscopy and biopsy, if available. As of March 11, 2021, when the first COVID-19 infection case was seen in Turkey, patients were contacted by phone or control examinations were carried out bimonthly. During the visits, laboratory tests and clinical conditions of these patients were recorded prior to pandemic emerging. In Turkey, all doctors can access official records for COVID-19 testing and view test positivity. Patients were carefully questioned for COVID-19, both from these official sources and in routine follow-up interviews with patients.

In the early stages of the pandemic, COVID-19 PCR testing was performed on individuals with at least one symptom. Diagnosis of COVID-19 was based on RT-PCR detection of SARS-CoV-2 RNA in nasopharyngeal swap specimens. Clinically, individuals with at least one symptom and a positive COVID-19 PCR test are considered COVID-19. Patients with COVID-19 PCR negative were not included in the study.

At the beginning of the pandemic, we had 159 cirrhosis patients registered in hospital database. Eleven of these patients had concomitant hepatocellular carcinoma (HCC). During the 10 months pandemic period, liver transplantation (LT) was performed in eight patients, five of whom were from living donors. No patients who received LT had COVID-19 infection before liver transplantation. Only one patient developed COVID-19 positivity on the 19th day of post-transplant. Except for prolonged COVID-19 PCR positivity, no significant COVID-19 related problem was observed in this patient. Patients who have baseline HCC and who received liver transplantation were excluded from the study.

In hospital’s clinical practice, cirrhosis patients come for routine follow-up controls bimonthly unless they have a special condition. The clinical evaluations and laboratory examinations of the patients at their visits just prior to tight lockdown applied country-wide were recorded. Most patients did not come to their follow-ups, especially during the strict lockdown practices, and they were contacted by telephone. With the loosening of restrictions in July 2020, which was called the normalization process, patients started to come to the hospital and their follow-up was carried out effectively. During this 10 months period, the clinical conditions of the patients were observed, and the course of the patients diagnosed with COVID-19 was closely followed. Of 140 patients with cirrhosis, 36 patients were diagnosed with COVID-19 infection in 10 months follow-up. In Turkey, patients exhibiting typical symptoms and signs for COVID-19 and those they had close contact with were tested for COVID-19.

### Data collection

Liver functions of all patients before and at the end of a 10 months pandemic period were evaluated. Age, gender, cirrhosis etiology, comorbidities such as diabetes-coronary artery disease-chronic kidney disease, laboratory tests [Alanine aminotransferase (ALT)-Aspartate aminotransferase (AST)-Gamma glutamyl transferase (GGT)-bilirubin level, international normalized ratio (INR), creatinine, sodium (Na) and platelets] were recorded in all patients. MELD-Na and Child- Turcotte-Pugh (CTP) scores were also calculated (Table 1 presented below shows the baseline clinical characteristics of all patients).

**TABLE 1:** Baseline clinical characteristics of the patients in the study group.

Characteristics	Patients (*n*: 140)
Age (years)	56 (18–89)
Gender	71 F/69 M
**Etiology of cirrhosis**	
NASH-related	36
Viral hepatitis-related	36
Autoimmune-related	16
Alcohol-related	15
Wilson disease	7
Budd-chiari syndrome	7
Cryptogenic or others (congenital hepatic fibrosis, cystic fibrosis, hemochromatosis etc.)	23
Diabetes mellitus	49 (35%)
Coronary artery disease (CAD)	13 (9%)
Chronic renal disease (CRD)	6 (4%)
Charlson comorbidity index (CCI)	4 (1–11)
Current or former smoker	51 (36%)
CTP-A/B/C	85/44/11
MELD-Na	11 (6–25)

### Statistics

Analysis of data was done by using the SPPS software version 21 (SPSS, Inc.,Chicago, IL, USA). Categorical variables are given as number of cases or percentages. Mean ± standard deviation and median values (minimum–maximum) were used for continuous variables. The Chi-squared test was used to compare two categorical variables. For continuous variables, non-parametric Mann Whitney U test was performed. Univariate and multivariate logistic regression models were used to evaluate the relationship between the various variables and clinical outcome. Odds ratios with their 95% confidence intervals (CI), along with corresponding *p*-values are calculated.

## Results

### Characteristics of patients

The median age of the patient group (71 F/69 M) was 56 and 35% were diabetic. In our practice, comorbidities of all cirrhotic patients are recorded at visits, and Charlson Comorbidity Index (CCI) scores of all patients are calculated. The median CCI score in our patient group was 4 (1–11). The general clinical characteristics of the patient groups are summarized in Table 1. When Cirrhosis (+)/COVID-19 (–) and Cirrhosis (+)/COVID-19 (+) patient groups were compared based on age, gender, baseline MELD score and CTP status of both patient groups did not differ. Table 2 (presented below), on the other hand, shows the baseline clinical features of both patient groups. The etiologies of cirrhosis patients with COVID-19 infection (*n*: 36) were as follows: HBV ± HDV infection (*n*: 10), Non-alcoholic steatohepatitis (*n*: 9), autoimmune liver diseases (*n*: 5), Budd-Chiari syndrome (*n*: 3), HCV (*n*: 2), congenital hepatic fibrosis (*n*: 2), alcoholic cirrhosis (*n*: 1), Wilson’s disease (*n*: 1), cryptogenic (*n*: 3).

**TABLE 2:** Baseline clinical and laboratory parameters of cirrhosis (+)/COVID-19 (+) and cirrhosis (+)/COVID-19 (–) patient groups.

	Cirrhosis (+)/COVID-19 (–) (*n*: 104)	Cirrhosis (+)/COVID-19 (+) (*n*: 36)	*P*
Age (years)	52 ± 16	56 ± 13	0.18
Gender	53 M/51 F	16 M/20 F	0.56
ALT	30 (7–291)	26 (8–112)	0.39
AST	40 (14–195)	38 (14–114)	0.63
GGT	64 (16–3470)	64 (12–220)	0.42
T Bilirubin	1.3 (0.29–9.36)	1.1 (0.45–8.32)	0.40
Platelet count (x10^9^/L)	98 ± 65	108 ± 76	0.61
INR	1.25 ± 0.26	1.26 ± 0.36	0.50
Creatinine	0.77 ± 0.45	0.74 ± 0.25	0.85
Albumin	3.69 (2.2–4.63)	3.65 (2.27–4.82)	0.99
CCI	4 (1–10)	5 (3–11)	0.40
MELD-Na	12.7 ± 4.35	11.8 ± 4.4	0.32
CTP score	CTP-A: 60 (57%) CTP-B: 36 (35%) CTP-C: 8 (8%)	CTP-A: 25 (69%) CTP-B: 8 (23%) CTP-C: 3 (8%)	0.33
DM	37%	30%	0.54
KAH	8.6%	13.8%	0.35
CRD	4.8%	2.7%	0.9

The main symptoms of the patients at the time of diagnosis were fever (55%), dyspnea (40%), myalgia and malaise (27%), cough (22%), gastrointestinal symptoms such as nausea-vomiting, diarrhea, abdominal pain (11%), and loss of sense of taste and smell (11%). In the course of COVID-19 infection, 16 patients with mild symptoms and signs were followed up at home. The remaining 20 patients needed hospitalization, and 12 of these patients were followed up in the intensive care unit. Twenty-seven of the patients received favipiravir therapy for COVID-19 infection. While four patients were receiving hydroxychloroquine treatment, five patients were followed up without treatment. Twenty-three of the patients used low molecular weight heparin therapy.

### Clinical course of patients

In our cirrhosis cohort, 11 patients died at 10 months follow-up. 5 (4.8%) of 104 patients in the Cirrhosis (+)/COVID-19 (–) patient group and 6 (16.6%) of 36 patients in the Cirrhosis (+)/COVID-19 (+) patient group died. The mortality rate in patients with COVID-19 infection is significantly higher than in other patients (*p*: 0.03). When the patients who died and those who did not die were compared, it was detected that the mean total bilirubin, MELD-Na scores of the patients who died were higher and the serum albumin level was lower. Table 3 compares the characteristics of patients with and without death shows that nine of the patients who died were initially CTP-B and C. In multivariate analysis, having had a COVID-19 infection [6.45 (1.43–29.4); *p*: 0.015], higher MELD-Na score [1.35 (1.18–1.60); *p*: 0.001] and higher CCI score [1.65 (1.14–2.39); *p*: 0.008] were found to be independent predictors of mortality.

**TABLE 3:** Comparison of the characteristics of cirrhosis patients with and without death.

	Univariate analysis			Multivariate analysis	
	Non-survivors (*n*: 11)	Survivors (*n*: 129)	*P*	OR (95% CI)	*P*
Age (years)	61 ± 12	52 ± 15	0.05	1.03 (0.95–1.11)	0.45
Gender	3 M/8 F	66 M/63 F	0.20		
ALT	28 (7–291)	30 (19–78)	0.59		
AST	51 (26–105)	38 (14–195)	0.30		
GGT	46 (20–157)	64 (12–3470)	0.57		
T Bilirubin	1.93 (0.77–9.36)	1.15 (0.29–8.32)	0.007	1.23 (0.76–1.96)	0.38
Trombosit (x10^9/^/L)	86 ± 38	109 ± 67	0.27		
INR	1.30 ± 0.25	1.25 ± 0.29	0.60		
Creatinine	0.74 ± 0.34	0.76 ± 0.41	0.87		
Albumin	2.79 (2.03–4.04)	3.75 (1.99–4.82)	0.001	0.26 (0.06–1.00)	0.06
CCI	5 (3–11)	4 (1–10)	0.05	1.65 (1.14–2.39)	0.008
MELD-Na	16.3 ± 5.8	12.1 ± 4.0	0.02	1.35 (1.18–1.60)	0.001
CTP score	CTP-A: 2 CTP-B: 5 CTP-C: 4	CTP-A: 83 CTP-B: 39 CTP-C: 7	0.01		
DM	3/11	47/129	0.74		
KAH	1/11	13/129	0.91		
CRD	1/11	5/129	0.39		
COVID-19 (+)	6/11	30/129	0.03	6.45 (1.43–29.4)	0.015

When the change in MELD-Na scores (delta MELD-Na score) at the beginning and at the end of the 10 months follow-up were evaluated after excluding patients who died, it was observed that the MELD-Na score in Cirrhosis (+)/COVID-19 (+) patients increased significantly compared to Cirrhosis (+)/COVID-19 (–) patients (-0.03 ± 3.28 vs. 1.55 ± 4.5; *p*: 0.045).

In fatal Cirrhosis (+)/COVID-19 (+) cases, one patient had CTP-A, three patients had CTP-B, and two patients had CTP-C cirrhosis at the beginning of the pandemic. One patient with CTP-A died from respiratory failure due to COVID-19, while they were stable in terms of liver disease. This patient did not develop a significant liver decompensation during their intensive care follow-up. While two patients with CTP-B and C died due to respiratory failure, three patients died because of hepatic decompensation and acute-on chronic liver failure (ACLF) after COVID-19 infection. In cirrhosis (+)/COVID-19 (–) cases (*n*: 104), out of five patients who died, one patient was CTP-A, two patients were CTP-B, and two patients were CTP-C at the beginning of pandemic, respectively. All patients in this group died secondary to natural course of cirrhosis (variceal bleeding, ACLF development, kidney failure, infections, etc.).

### Vaccination status of the patients

When the COVID-19 vaccination status was examined in our cirrhosis patient group (*n*: 129), it was found that 14 patients (10.8%) did not receive any vaccine in the following 10 months, and five patients (3.8%) received only a single dose of CoronaVac vaccine. It was observed that all other patients had at least two doses of CoronaVac or BNT162b2 mRNA COVID-19 (BioNTech) vaccine. Since the vaccination process was started with the CoronaVac vaccine in Turkey, the first two doses of the vaccine were administered as CoronaVac in most of the patients. While 45 of these patients (received two or three doses of CoronaVac vaccine, 33 patients received two doses of CoronaVac as a third dose of BioNTech, and 26 patients received at least two doses of BioNTech vaccine. Vaccination information of six patients could not be reached.

### Clinical course after starting COVID-19 vaccination

After the 10 months pandemic period, with the start of vaccination, 11 (8.5%) of 129 patients who were followed up for about 10 months developed COVID-19. Only one patient developed severe pneumonia and subsequently died. It was learned that this 42 years-old patient, who was followed up for cryptogenic cirrhosis, was unvaccinated. Other 10 patients who had COVID-19 had at least two doses of vaccine. None of these patients developed COVID-19-related death or serious illness. In this process, 10 patients died due to cirrhosis and its complications.

## Discussion

This study investigates the effects of the COVID-19 infection on the natural course of cirrhosis as our own cohort before and after effective vaccination. In our well-documented cirrhosis patient group consisting of 140 patients, the clinical conditions before March 11, 2020, when the first COVID-19 case was seen in Turkey, and the clinical conditions after 10 months pandemic period were compared. In this period, mortality rates and clinical progression rates of patients with and without COVID were compared. There was a total of 11 deaths in our series of cirrhotic patients. The mortality rate in those with documented COVID-19 infection was determined as 16.6%, this rate was 4.8% in the patient group who did not have a documented COVID-19 infection. Looking at non-COVID-19 deaths, deaths due to decompensation and ACLF development were observed in the natural course of the disease. In COVID-related deaths (*n*: 6), four patients died from respiratory failure while their liver diseases were under control. The presence of COVID-19 infection was identified as an independent predictor of mortality in multivariate analysis with an OR of 6.45. Other independent predictors for mortality were higher MELD-Na score [1.35 (1.18–1.60); *p*: 0.001] and higher CCI score [1.65 (1.14–2.39); *p*: 0.008]. There was an increase in the number of patients who progressed to CHILD-B and CHILD-C during the 10 months-pandemic in both groups (Figure 1). According to these findings, although it is difficult to state that there is an increase in decompensation complications in Cirrhosis (+)/COVID-19 (+) patients when compared to Cirrhosis (+)/COVID-19 (–) patients during the pandemic, when the pre-pandemic and post-pandemic MELD scores are examined, it was found that MELD scores increased significantly in Cirrhosis (+)/COVID-19 (+) patients. Follow-up continued in the cirrhotic patient group with the initiation of vaccination against COVID-19 (January 14, 2021). Patients were advised to get their vaccinations. The first vaccine administered in Turkey was the inactive CoronaVac vaccine. In the following period, BNT162b2 mRNA COVID-19 (BioNTech) vaccine has also been used in Turkey. During 10 months follow-up period, no COVID-19 related patient deaths were observed in the cirrhotic patient group with vaccination. Only one unvaccinated cirrhosis patient had a COVID-19 infection-related death. This study shows that inactive CoronaVac and BNT162b2 mRNA COVID-19 (BioNTech) vaccine significantly prevent death and severe disease in cirrhosis patients.

In the past year, the devastating effects of the COVID-19 pandemic have been and are being experienced all over the world. Mortality related factors in COVID-19 infection have been shown in many studies. Advancing age, male sex, presence of comorbidities including diabetes, malignancy, hypertension and heart diseases have been shown to be associated with severe COVID-19 infection (8–11). The course of the disease in chronic liver disease has also been shown in many studies. It is now known that the liver is one of the organs seriously affected by this disease. The effect of SARS-Cov-2 on the liver has not been fully elucidated. Although various studies have been conducted on the hepatotropic effects of the virus to date, the evidence obtained is limited. Studies conducted in healthy individuals with normal livers have shown that the virus can cause liver damage with a direct cytopathic effect through ACE-2 receptors in hepatocytes and cholangiocytes. In addition, uncontrolled immune response due to COVID-19 infection, microvascular and macrovascular thrombosis, and drug-induced liver injury have also been suggested as other mechanisms (12, 13).

Studies conducted in patients with known liver disease were mostly conducted on cirrhosis. The mechanisms suggested in these cases are mostly related to cirrhosis-associated immune dysfunction. The results obtained in studies on this subject differ for various reasons. One of the studies reporting a high mortality rate is the large international registry study conducted by Marjot et al. (14) during the first months of the pandemic. In this study, the clinical outcomes of 745 CLD patients (386 with cirrhosis and 359 without cirrhosis) who had COVID-19 infection were evaluated, and a mortality rate of 32% in CLD patients with cirrhosis and 8% in CLD patients without cirrhosis was found (14). Another study in which a high mortality rate was reported is a study conducted in Italy as pandemic commenced. In this study, 50 cirrhosis patients with SARS-CoV-2 infection were evaluated and it was reported that 17 (34%) of the patients died within 10 days after the diagnosis of COVID-19 (5). In another retrospective multicenter cohort study, hospitalized cirrhosis (+) COVID-19 (+) patients were evaluated and mortality was found to be 30% in this patient group. In another study, overall all-cause mortality was found to be 14% in CLD patients and the presence of decompensated cirrhosis was found to be an independent predictor of mortality (15). According to the studies conducted, while COVID-19 infection progresses with 8% mortality in non-cirrhotic chronic liver disease, this rate is 22% in CHILD-A cirrhosis, 39% in CHILD-B cirrhosis, and 54% in CHILD-C cirrhosis. Especially in cases requiring intensive care, these rates are even higher (16). Studies on this subject in the literature are mostly studies conducted in the early periods of the COVID-19 pandemic. We think that it should be considered that the information about the COVID-19 pandemic and prevention measures is limited in this period. Likewise, in the early periods of the pandemic, the health-care system had almost reached the point of blockage in many places and the health service was disrupted. In studies conducted in this period, it is expected that such high mortality rates were obtained in the cirrhosis patient group.

One of the remarkable findings in our patient group is that almost a quarter of them were infected with COVID-19, despite the patient cohort were being well-informed and had strictly adhered to precautions in the pre-vaccine period. Cirrhosis has been shown in a recent study as a risk factor for short-term mortality (17). COVID-19 infection was fatal in 16% of our patients. According to the multivariate analysis, it is revealed that the risk of death in cirrhosis patients with COVID-19 infection increases approximately six times during 10 months follow-up. In our patient group, the mortality rate in cirrhosis cases was lower when compared with other studies. Although the low death rate can be loosely explained by the fact that much of the patient group is composed of CTP-A cirrhotic patients, the main reason that one can explain the low mortality rate is the frequent testing of possible contact cases in Turkey. In this way, asymptomatic COVID-19 cases could also be diagnosed, and the course of the disease was milder in these patients. COVID-19 vaccination in Turkey was initiated with healthcare workers on January 14, 2021. The effectiveness of currently used vaccines in preventing the disease has been demonstrated in many studies (6, 7). The efficacy of vaccines in chronic liver diseases has also been demonstrated in various studies. In a study conducted in chronic hepatitis B patients, it was observed that inactive COVID-19 vaccines developed an effective antibody response in this patient group, including those with cirrhosis (18). In a study conducted in a group of patients administered mRNA and vector-based vaccines, the antibody responses of cirrhotic patients were roughly similar to the control group (19). In one study, a weak antibody response was observed in 24% of chronic hepatitis with or without cirrhosis (20). Little is known about the clinical significance of a weak antibody response. In our study, no COVID-19-related death or severe illness was observed in vaccinated individuals with the initiation of vaccination in Turkey. Only one unvaccinated cirrhosis patient died due to severe pneumonia and sepsis associated with COVID-19. The strengths of our study are that it examined the natural history of a well-documented and well-followed cirrhosis patient group during the 10 months pandemic period and 10 months after vaccination started. The strengths of the study certainly are that all patients were followed closely and that information on COVID-19 infection and their survival could be obtained reliably. The fact that the antibody response of the vaccinated patients which could not be evaluated can be considered as a limitation of the study. In conclusion, this study clearly showed that COVID-19 infection in the pre-vaccine period caused high mortality in cirrhotic patients and that no negative effect of COVID-19 infection on the natural history of cirrhosis was observed with vaccination.

## Data availability statement

The raw data supporting the conclusions of this article will be made available by the authors, without undue reservation.

## Ethics statement

The studies involving human participants were reviewed and approved by Hacettepe University Ethics Commitee (GO 21/561). The patients/participants provided their written informed consent to participate in this study.

## Author contributions

OK designed the study, was actively involved in acquisition of data and in the analysis, interpretation of data, supervised study related procedures, and wrote the first draft of the manuscript. HO and TS were actively involved in acquisition of data and critical revision of the manuscript. TK and EP designed the study, were actively involved in acquisition of data and in the analysis, interpretation of data, supervised study related procedures, and critical revision of the manuscript. All authors contributed to the article and approved the submitted version.
